# Using Physiologically-Based Pharmacokinetic Models to Incorporate Chemical and Non-Chemical Stressors into Cumulative Risk Assessment: A Case Study of Pesticide Exposures

**DOI:** 10.3390/ijerph9051971

**Published:** 2012-05-22

**Authors:** Susan C. Wason, Thomas J. Smith, Melissa J. Perry, Jonathan I. Levy

**Affiliations:** 1 Department of Environmental Health, Harvard School of Public Health, 401 Park Drive West, Boston, MA 02215, USA; Email: tsmith@hsph.harvard.edu (T.J.S.); mperry@gwu.edu (M.J.P.); jonlevy@bu.edu (J.I.L.); 2 Department of Environmental and Occupational Health, School of Public Health and Health Service, George Washington University, 2300 Eye St. NW, Washington, DC 20037, USA; 3 Department of Environmental Health, Boston University School of Public Health, 715 Albany St., Boston, MA 02118, USA

**Keywords:** cumulative exposure, risk assessment, pesticides, health disparities, diet

## Abstract

Cumulative risk assessment has been proposed as an approach to evaluate the health risks associated with simultaneous exposure to multiple chemical and non-chemical stressors. Physiologically based pharmacokinetic/pharmacodynamic (PBPK/PD) models can allow for the inclusion and evaluation of multiple stressors, including non-chemical stressors, but studies have not leveraged PBPK/PD models to jointly consider these disparate exposures in a cumulative risk context. In this study, we focused on exposures to organophosphate (OP) pesticides for children in urban low-income environments, where these children would be simultaneously exposed to other pesticides (including pyrethroids) and non-chemical stressors that may modify the effects of these exposures (including diet). We developed a methodological framework to evaluate chemical and non-chemical stressor impacts on OPs, utilizing an existing PBPK/PD model for chlorpyrifos. We evaluated population-specific stressors that would influence OP doses or acetylcholinesterase (AChE) inhibition, the relevant PD outcome. We incorporated the impact of simultaneous exposure to pyrethroids and dietary factors on OP dose through the compartments of metabolism and PD outcome within the PBPK model, and simulated combinations of stressors across multiple exposure ranges and potential body weights. Our analyses demonstrated that both chemical and non-chemical stressors can influence the health implications of OP exposures, with up to 5-fold variability in AChE inhibition across combinations of stressor values for a given OP dose. We demonstrate an approach for modeling OP risks in the presence of other population-specific environmental stressors, providing insight about co-exposures and variability factors that most impact OP health risks and contribute to children’s cumulative health risk from pesticides. More generally, this framework can be used to inform cumulative risk assessment for any compound impacted by chemical and non-chemical stressors through metabolism or PD outcomes.

## 1. Introduction

Cumulative risk assessment has recently emerged as an area of interest among regulators as well as stakeholders concerned about environmental justice [1,2,3]. The U.S. EPA’s Framework for Cumulative Risk Assessment defines cumulative risk formally as “the combined risks from aggregate exposures to multiple agents or stressors” [4]. Of note, the EPA considers cumulative risk assessment to include both chemical and non-chemical stressors, the latter of which may potentially include (but not be limited to) low income, low community property values, limited access to health care, psychosocial stress, and other stressors not commonly within the purview of EPA decision-making.

Despite the inclusion of non-chemical stressors in the definition of cumulative risk, cumulative risk assessments to date have typically ignored those stressors [5]. This is largely because toxicological studies do not have the capacity to consider most non-chemical stressors, as well as because of the limited availability of epidemiological evidence. However, a recent report evaluating risk assessment methods [6] reinforced the priority that needs to be placed on evaluating risks from multiple stressors simultaneously, with a particular emphasis on identifying how multiple chemical and non-chemical stressors impact individual and population health. 

Conceptual frameworks have been developed for cumulative risk assessments that capture chemical and non-chemical stressors [7], but such frameworks have not been linked with the methods commonly used for modeling exposures and health outcomes within risk assessment. In particular, there are limited methods to date to evaluate the impacts of chemical and non-chemical stressors on the internal doses or resulting health effects of an environmental toxicant.

Pesticides have been at the forefront of the cumulative risk discussion, based on the Food Quality Protection Act of 1996, which specifically mandates that pesticides with a common mechanism of action be evaluated for their cumulative health risks [8]. Cumulative risk assessments have been conducted for organophosphate (OP) pesticides, which as a group have a common primary mechanism of action, acetylcholinesterase (AChE) inhibition [9,10,11]. These assessments have included multiple OPs and exposure pathways, capturing one key dimension of modeling neurocognitive effects, but did not consider other potential stressors with similar mechanisms or that would impact the metabolism of OPs or their mechanism of action. Non-chemical physiological and psychosocial stresses can have indirect biochemical and neurological effects similar to those of the OPs. This issue may be particularly salient for children in low-income urban environments, who have been shown to have simultaneous exposure to multiple pesticides [12] and who exhibit high levels of three important non-chemical stressors: obesity, inadequate nutrition, and psychosocial stress. A recent study in urban public housing [13] found 56% of children to be overweight, with 41% of caregivers not allowing their children to play outside due to neighborhood violence and 84% of caregivers of children under age 8 reporting fear of violence. However, no studies have formally considered how these stressors could influence the health risks associated with OP exposures.

Within this study, our aim was to develop a theoretical risk framework for evaluating cumulative risk to OPs in the presence of other chemical and non-chemical stressors, using physiologically-based pharmacokinetic/pharmacodynamic (PBPK/PD) models to quantify the impacts of these stressors on OP internal dose and AChE inhibition. We developed methods for incorporating the biochemical effects of a subset of common non-chemical stressors affecting metabolism and the pharmacodynamic outcome. We simulated internal doses and AChE inhibition for children characteristic of an urban low-income environment and discuss the relative impacts of multiple stressors on OP doses and factors that contribute most to increased risk of AChE inhibition amongst this population. The theoretical framework developed here, with OPs as an illustrative example, can be utilized for any chemical that may be impacted by stressors through metabolism or mechanism of action, and provides a first effort at a methodological framework to quantify the cumulative impact of chemical and non-chemical stressors on an individual’s health risk.

## 2. Methods

### 2.1. Cumulative Risk Framework Development

**Figure 1:**
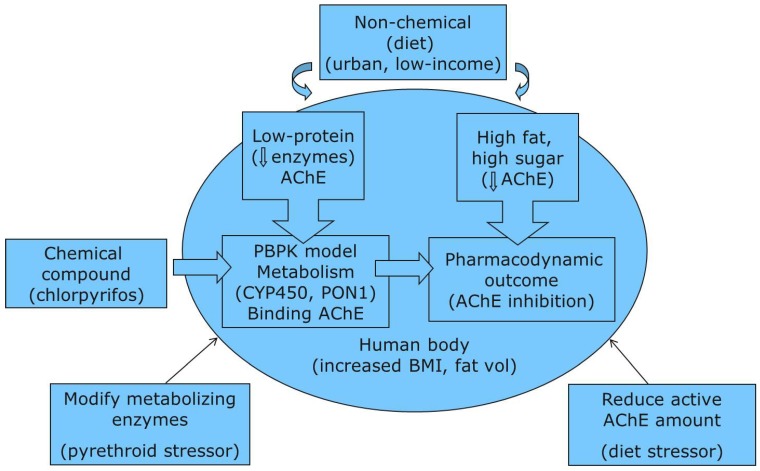
Diagram of risk framework using PBPK/PD model application for OPs with chemical and non-chemical stressors (Parentheses depict specifics for this example).

We developed a framework for cumulative risk assessment that relies on an understanding of the physiologic processes that lead to a critical health outcome, such as AChE inhibition. Figure 1 shows a diagram of our conceptual framework using an OP, chlorpyrifos (CPF), and exposure and stressors for urban low-income children as an example (described in detail in sections to follow). In this framework approach, biochemicals, metabolic processes and target tissues are identified that would be affected by exposure to OPs or stressors, including the general pool of enzyme proteins, enzymes specific to OP metabolism, and the pool of AChE enzyme, particularly in the brain. Specific stressors are identified that have direct effects on AChE (e.g., CPF-oxon metabolite) and that affect the general pool of enzyme proteins available for physiological needs, including AChE (e.g., diet). 

The elements of the framework are integrated by a PBPK/PD model to describe the internal relationships of tissue concentrations, enzymatic processes and the quantitative effects of chemical and non-chemical stressors, and quantification of a health relevant outcome, in this case changes in AChE inhibition. Thus, within the present study, framework development involved systematically evaluating which stressors were both relevant for our urban low-income population of children and could influence PBPK/PD model parameters. Specifically, we sought out evidence about stressors that would influence general physiological factors, or that would impact the metabolism of OPs and the percent AChE inhibition, both model components that would directly impact CPF-oxon doses or the health outcome. We then used the developed framework to infuse changes that would reflect CPF PBPK/PD model parameter impacts due to stressors relevant for our population with this example.

Within this study, though OP doses vary with age, gender, and other characteristics, we simulated sets of individuals with a fixed set of characteristics in order to isolate the impacts of the additional stressors of interest. We modeled 2-year-old females with a median height, noting that 2-year-olds will have greater enzyme levels than younger children. We performed our stressor simulations using a modified version of the CPF PBPK/PD model, previously developed by Timchalk *et al.* [14]. 

### 2.2. Evaluation of Additional Chemical Stressors

Both pyrethroids and OP pesticides were found frequently in the homes of children in the low-income population we use to generate our study population, with many homes having five or more pesticides across both groups [12]. OPs and pyrethroids are metabolized by similar CYP enzymes, with pyrethroids also being primarily detoxified by carboxylesterase enzymes (which are almost irreversibly bound to OPs). Significant associations were found between CPF metabolites and CYP3A4/5, CYP2C8, CYP2C19 and CYP1A2 [15]. Human CYP450 isoforms that showed activity toward multiple pyrethroids were CYP2C8, CYP2C9, CYP2C19, and CYP3A4 [16], indicating that an overlapping profile of metabolizing enzymes exists for the two groups. While OPs impact pyrethroid metabolism significantly, pyrethroids have been found to be weak inhibitors of OPs [17]. However, the combined exposure and shared metabolic enzymes indicate that pyrethroids also have the potential to have an effect on OP dosimetry. Other chemical stressors may have been present in the residential environment that could impact CPF or CPF-oxon doses, but they were unknown or not characterized for this population. No other chemicals were present to our knowledge that directly impacted AChE inhibition (e.g., carbamates). 

### 2.3. Evaluation of Non-Chemical Stressors

Within our literature review, we focused on non-chemical stressors that were relevant to low-income urban children, which would potentially influence the effects of OP exposure, and had sufficient data available to indicate how the PBPK/PD model should be modified. While there is insufficient information to understand the majority of the possible interactions that could occur between chemical and non-chemical stressors, we sought to capture how selected common non-chemical stressors might modify general physiological parameters (e.g., weight), metabolic parameters (e.g., V_max_, maximum velocity of reaction), and AChE inhibition (e.g., through estimating underlying AChE levels). 

A host of potential stressors relevant to urban or low-income children have been reported. Given our emphases listed above, we found the most consistent and relevant evidence to support psychosocial stress (often related to exposure to community violence) and diet as non-chemical stressors for this population. Stress and diet were thus further evaluated for their potential impacts on physiology, OP metabolism, and AChE inhibition. 

First considering psychosocial stress, studies confirmed its importance for a variety of health outcomes as well as consistently elevated rates in low-income urban populations [18,19]. Exposure to community violence represented a particular stressor within these communities [20,21,22]. Severe acute or chronic stress has been shown to impact important metabolic pathways, particularly causing hormonal changes that can lead to increased susceptibility to environmental toxicants [23,24]. However, evaluations of direct impacts of acute and repeated stress in animals prior to CPF exposure showed no impact on AChE inhibition [25]. Thus, although psychosocial stress is clearly important for numerous outcomes and likely impacts these exposures in some capacity, quantitative evidence did not presently exist to suggest a direct impact on either OP metabolism or AChE inhibition, and data regarding general impacts were not available that could be incorporated in this assessment. 

For diet, obesity rates are highest among low-income, minority, urban children [26], with a recent study in urban public housing [13] finding 56% of children to be overweight. Irigoyen *et al.* also found obesity percentages for urban low-income children ages 1–5 to be 7.5% (age 1), 20% (age 2), and 30% (ages 3–5) [26]. Increased weight leads to increased fat volumes, which could lead to prolonged exposure to OPs for obese children due to the high fat partition coefficients for OPs. Furthermore, low-income children were more likely to have a diet comprised of lower protein and a higher percentage of fat and sugars, with mean percentage of calories from carbohydrates, protein, and fat of 57%, 13%, and 32%, respectively, with between 69–94% not meeting the minimum number of servings of fruit, vegetables, grain, meat/poultry, and dairy [27]. These dietary impacts can lead to changes in important enzyme levels and metabolism of environmental toxicants [28]. Long-term high sugar, high fat diets have been directly associated with decreased levels of AChE activity [29], with low protein diets more generally associated with lower enzyme production levels [30,31]. Decreased AChE activity due to diet could add to the percent inhibition occurring due to OP exposure. Low enzyme production could lead to decreased CPF-oxon production, but also decreases in the PON1 enzymes needed to detoxify the CPF-oxon metabolite. Thus, the available evidence supported diet as having a direct impact on both metabolism and AChE inhibition, with obesity additionally influencing the PBPK/PD model structure and outputs. 

### 2.4. Simulation Approach

As a result of our review of stressors for urban low-income children, enzymatic impacts from concurrent pyrethroid exposure and physiological changes from dietary factors were included, with the resulting framework illustrated in Figure 1. The effects of pyrethroid exposure were incorporated through the metabolism compartment, with modifications in the V_max_ (maximum velocity of reaction) parameter values for the CYP and PON1 metabolizing enzymes. Dietary factor impacts were incorporated in several ways. First, elevated body mass index was incorporated through overall body weight and tissue volume modifications, in order to evaluate whether body weight, and in particular fat volume, influenced our findings. Dietary stressor impacts were also incorporated through the compartments of metabolism and the pharmacodynamic outcome. A low-protein diet reduces the formation rate of enzyme protein, which proportionally reduces V_max_, increasing sensitivity to OP exposure. Lower protein could also reduce metabolic formation of toxic OP metabolites. The pharmacodynamic outcome was incorporated through decreased AChE levels in the body due to a high fat/high sugar diet.

In all cases, data did not allow for precise estimation of the effect of the chemical or non-chemical stressor, but we developed approximations to capture the upper bound potential impacts of these stressors. Distributions of data for these stressors would be optimal to include in order to fully consider variability due to these stressors. However, incorporation of bounding estimates in the absence of available distributional data allows for an initial characterization of the likely importance of these stressors from a health relevant standpoint and demonstration of proof of concept of our theoretical framework. For alterations in the metabolism compartment, we assumed a theoretical value of 50% reduction in V_max_ values as a result of co-exposure to pyrethroids and decreased overall enzyme levels with a low-protein diet. Although exact effects of diet on metabolism have not been reported, studies have consistently shown that diet has a negative impact on overall enzyme levels [32,33,34]. Furthermore, a 50% reduction in V_max_ is plausible given ranges of V_max_ values that capture polymorphic enzymes and interindividual variability. Foxenberg *et al.* reported V_max_ values that spanned between 2–12 fold depending on the enzyme group, with CYP enzymes having a 4-fold V_max_ range for the 2-year-old age group (e.g., from half the standard reported value to 2× the reported value) [35].

Effects of body mass index were simulated by incorporating a range of plausible values, determined from the Center for Disease Control and Prevention’s clinical growth charts [36]. We focused on females age 1–2 (12–24 months) and considered children of median weight at 18 months (10.9 kg) as the comparison group. A lower bound weight was taken from the 5th percentile at 12.5 months (8.13 kg) and an upper bound weight was taken from the 95th percentile at 23.5 months (14.5 kg). In all cases, we considered a median height of 79.8 cm to allow for a range of body mass index values. This method created body mass index values well below the 5th and well above the 95th percentile, to examine the range of impact for this framework exercise. A long-term high fat/high sugar diet caused between 20–40% decreases in AChE activity for females in animal studies [29]. This potential decrease could reduce the amount of AChE available to break down acetylcholine, in combination with the reduction occurring through OP exposure. In order to quantify how much of an impact the diet stressor could have on AChE inhibition, we assumed the maximum reported decrease of 40% in AChE binding sites during the simulations.

With these parameter modifications in place, we ran a series of sensitivity analyses across selected CPF input exposure values from previous exposure modeling work. This study simulated dermal and ingestion exposures for young children from dust concentration measurements in their urban low-income residences [37,38] (median, 95th percentile, and maximum). We combine these estimates for 2-year-old children with varying combinations of stressors (*i.e.*, across weight values, with the pyrethroid chemical stressor, with the low protein, high fat and sugar diet stressor, and with both stressors). CPF doses were simulated for 21 days of exposure, in order for AChE inhibition to reach steady state. We used the models to estimate the theoretical CPF-oxon area-under-the-curve (AUC) in the brain tissues at day 21, along with the maximum value percent AChE inhibition at steady state associated with CPF-oxon concentrations. Maximum value percent inhibition at day 21 is presented here to represent the upper bound value that could occur in each sensitivity analysis. In each case, we compare children with additional chemical and non-chemical stressors with the child within their exposure range with normal parameters as a baseline. We determined which factor or combination of factors most significantly influence percent AChE inhibition in the brain. 

## 3. Results

First considering a child at median exposure, the CPF-oxon brain AUC values and corresponding maximum percent AChE inhibition in the brain varied significantly across stressor simulations, with percent AChE inhibition varying from 14% less to 5 times more than seen for the baseline child (Table 1). The highest percent inhibition values corresponded with children with the 95th percentile weight and both the pyrethroid and diet stressors (Table 1). While the combination of stressors yielded the greatest effect, all stressors individually contributed to increases in percent AChE inhibition. For example, the increased body mass index contributed to a roughly proportionate increase in percent AChE inhibition. The reduced V_max_ approximately doubled AChE inhibition given normal AChE binding sites, but displayed a less than proportionate increase given decreased binding sites. The AChE binding site decreases from diet contributed an approximate factor of 2–4 increase in inhibition across other parameter values. At higher levels of exposure, the patterns are similar, though with some modest differences in the individual and joint contributions of factors (Table 1). As expected, percent AChE inhibition increased with exposure level across simulations. In each case, only the child with low body weight and normal levels of V_max_ and AChE binding sites demonstrated lower percent AChE inhibition than the baseline child, with approximate 20% reductions. Additionally, in each case, the child with high body weight and both the pyrethroid and diet stressors exhibited inhibition approximately 2.5 times higher than the level for the baseline child (a lower ratio than seen for the children at median exposure). Each of the stressors made a significant contribution, though at the higher exposure levels, decreased binding sites had a lower relative influence than at the lower exposure levels, suggesting that enzyme saturation may occur at the higher exposure levels. At the highest exposure level and combination of stressors, the percent AChE inhibition reached a maximum value of 0.148%, substantially greater than for the child at median exposure without additional stressors.

**Table 1:** Percent AChE inhibition changes in the brain after 21 day CPF exposure with inclusion of a chemical and non-chemical stressor.

Exposure (μg/kg/day)	Weight	V_max_	AChE binding sites	AUC CPF-oxon brain (μg/kg/day)	% AChE inhibition brain(Maximum)	Ratio - % inhibition in simulated child/ % inhibition in comparison child
Median (0.075 derm, 0.003 ing)	median(10.9 kg)	normal *	normal	0.056	0.00028	Median child
	5% (8.13 kg)	normal	normal	0.060	0.00024	0.86
	95% (14.5 kg)	normal	normal	0.050	0.00036	1.29
	median	↓ 50%	normal	0.111	0.00057	2.04
	5%	↓ 50%	normal	0.119	0.00048	1.71
	95%	↓ 50%	normal	0.100	0.00069	2.46
	median	normal	↓ 40%	0.056	0.00100	3.57
	5%	normal	↓ 40%	0.060	0.00090	3.21
	95%	normal	↓ 40%	0.050	0.0011	3.93
	median	↓ 50%	↓ 40%	0.111	0.0013	4.64
	5%	↓ 50%	↓ 40%	0.119	0.0012	4.29
	95%	↓ 50%	↓ 40%	0.100	0.0014	5.00
95th percentile (0.876 derm, 0.417 ing)	median	normal	normal	0.631	0.0034	95th % child
	5%	normal	normal	0.618	0.0027	0.79
	95%	normal	normal	0.615	0.0041	1.21
	median	↓ 50%	normal	1.26	0.0068	2.00
	5%	↓ 50%	normal	1.23	0.0055	1.62
	95%	↓ 50%	normal	1.23	0.0081	2.38
	median	normal	↓ 40%	0.631	0.0046	1.35
	5%	normal	↓ 40%	0.618	0.0038	1.12
	95%	normal	↓ 40%	0.615	0.0055	1.62
	median	↓ 50%	↓ 40%	1.26	0.0074	2.18
	5%	↓ 50%	↓ 40%	1.23	0.0062	1.82
	95%	↓ 50%	↓ 40%	1.23	0.0088	2.59
Maximum (15.9 derm, 0.820 ing)	median	normal	normal	10.8	0.0615	Max child
	5%	normal	normal	9.69	0.0501	0.81
	95%	normal	normal	9.62	0.0738	1.20
	median	↓ 50%	normal	21.5	0.1230	2.00
	5%	↓ 50%	normal	19.3	0.1000	1.63
	95%	↓ 50%	normal	19.2	0.1470	2.39
	median	normal	↓ 40%	10.8	0.0720	1.17
	5%	normal	↓ 40%	9.69	0.0580	0.94
	95%	normal	↓ 40%	9.62	0.0880	1.43
	median	↓ 50%	↓ 40%	21.5	0.1230	2.00
	5%	↓ 50%	↓ 40%	19.3	0.1000	1.63
	95%	↓ 50%	↓ 40%	19.2	0.1480	2.41

* Normal values still scaled by weight, then modified; derm = dermal, ing = ingestion.

## 4. Discussion and Conclusions

In this study, we have developed and applied a framework (shown in Figure 1) for using PBPK/PD modeling to quantitatively evaluate the impacts of chemical and non-chemical stressors on an individual chemical exposure and corresponding potential health impact. Within our simulations, focused on CPF exposures, doses and percent AChE inhibition varied considerably (up to a factor of five) across stressor factor combinations. Even given the limited information available to estimate effects within a PBPK/PD framework, our approach allowed for characterization of relative impacts of multiple stressors on OP doses and factors that contribute most to increased risk of AChE inhibition amongst this simulated population. 

We concluded in this example that high body mass index and physiological impacts from pyrethroid and diet stressors would impact the amount of AChE inhibition associated with CPF exposures. This combination of stressors is likely to be present for young children living in an urban low-income environment. While variability in exposure inputs would tend to dominate the effects of these stressors [37,38], omission of these and other stressors may underestimate the effects of pesticides in low-income communities. Some non-linearities were seen at the higher exposure levels with the inclusion of the diet stressor, which decreases available AChE levels. In comparison to the linear relationship seen with increased percent inhibition as stressors were added to the analyses at the median exposure levels, the relationship at the higher exposure levels with the inclusion of diet was non-linear, suggesting that enzyme saturation may occur at these exposure levels. Our study suggests the importance of a systematic methodology to consider the effects of numerous chemical and non-chemical stressors within PBPK/PD modeling, as an approach for enhancing cumulative risk assessment and providing unbiased health risk estimates. 

There are some clear limitations in our analysis, even given the fact that we presented an intentionally stylized example. First, even with the combination of stressors at the highest level of exposure, the percent AChE inhibition would seem to indicate limited risk, which would reduce the importance of incorporating various stressors. Studies in the literature indicate that >20% inhibition is generally considered as a level of concern, as compared with our maximum level of 0.148%, although the threshold value may be lower for developing children [9]. In other words, the variability in this risk-relevant metric would not likely correspond with detectable variability in risk. However, our exposure inputs only included non-dietary routes of exposure, and inclusion of additional routes of exposure as well as additional pesticides and non-chemical stressors may lead to more significant percent AChE inhibition estimates. More generally, our goal was not to comprehensively determine exposures and corresponding health risks, but to suggest a methodology by which non-chemical stressors could be introduced into the cumulative risk paradigm, and our methods could be applied in many other contexts. 

In addition, the proposed framework may not be applicable in the near term for many compounds, given limitations in the number of currently available PBPK/PD models, especially given the desire for models with the pharmacodynamic component that allows for evaluation of a health relevant outcome. However, this proof of concept application coupled with growing interest in cumulative risk assessment may stimulate the development of novel PBPK/PD models with cumulative risk applications in mind, or the consideration of how various chemical and non-chemical stressors may influence metabolic processes or pharmacodynamic outcomes in existing models. This is supported by a recent article by Tan *et al.*, which considered approaches for evaluating interactions between multiple chemical stressors with PBPK/PD modeling and highlighted the overall utility of computational modeling for cumulative risk assessment [39]. Efforts are also ongoing presently to develop a family PBPK model for the pyrethroid pesticides, building upon deltamethrin models developed and refined by Mirfazaelian *et al.* [40] and Godin *et al.* [41], respectfully, which could potentially allow for evaluation of chemical and non-chemical stressor impacts on this widely used pesticide group. As mentioned previously, OPs almost irreversibly inhibit the major metabolizing enzymes for pyrethroids, carboxylesterases, leading to increased toxicity from the parent compound that could extend to human health effects [42]. If OPs were incorporated as a chemical stressor for pyrethroids, the impact of OPs on pyrethroid toxicity could be evaluated using this framework, providing great insight into the magnitude of health impact changes occurring as a result of their combined exposures. 

An additional limitation stems from the fact that we were limited in available data regarding stressors, both for stressors included in this study and those not included, since we could only capture stressors with documented impacts on metabolism or that have been previously shown to directly impact the pharmacodynamic outcome. Other stressors, including but not limited to psychosocial stress, likely impact these physiological systems and outcomes in a manner that may lead to a relevant health impact; however, data did not exist at the time of our investigation to adequately understand or quantify these impacts in a PBPK/PD modeling framework. Data were also limited regarding the stressors that were incorporated. Additional data are necessary in order to utilize representative data distributions to capture the full range of variability that may occur due to these stressors. An intentionally stylized approach with bounding calculations for stressor data was applied in this study, with the aim that the theoretical framework concept derived here could be further validated and enhanced as more data becomes available. Propagating uncertainty and variability throughout the analysis would allow for refinement of the potential health impact of these stressors and evaluation of the sources and magnitude of uncertainty and variability. We recommend additional studies and data collection oriented around the effects of these non-chemical stressors on specific PBPK/PD model parameters. Future studies should evaluate stressor data variability and uncertainty and report these values. More generally, while our inclusion of stressors is clearly not comprehensive, the stressors addressed within our application allow us to understand the potential magnitude of impact that a stressor of this kind may have on a specific chemical exposure. 

In spite of these limitations, our work offers a new methodological framework within which chemical and non-chemical stressors can be jointly considered in the emerging area of cumulative risk assessment. The theoretical framework developed here, with OPs as an illustrative example, can be utilized for any chemical that may be impacted by stressors through metabolism or mechanism of action. This structure can be utilized to evaluate the impacts of multiple chemical and non-chemical stressors simultaneously on an individual chemical. This work could also be extrapolated to include multiple primary compounds within the same chemical family, or with the same mode of action (e.g., multiple OPs). The framework provided herein includes both a systematic approach to evaluate potential stressors for inclusion in the assessment as well as quantitative methods to assess the impact of included stressors on a health relevant outcome, which can help determine which stressors may be important to include from a health-relevant standpoint.
